# Special Issue: Firefighters’ Occupational Exposures and Health Risks

**DOI:** 10.3390/toxics13050343

**Published:** 2025-04-26

**Authors:** Maria Helena Guerra Andersen, Anne Thoustrup Saber

**Affiliations:** The National Research Centre for the Working Environment, Lersø Parkallé 105, 2100 Copenhagen, Denmark

## 1. Introduction

Firefighters are occupationally exposed to a complex mixture of hazardous agents. Beyond the immediate risks of injuries and accidents, they are routinely exposed to smoke and soot, toxic combustion byproducts, and other harmful chemicals, as well as physical exhaustion, hyperthermia, dehydration, night work, noise, and mental stress. These exposures from firefighting have also been associated with cancer [1], cardiovascular disease [2,3], and adverse reproductive effects [4,5]. The list of chemicals that can make up fire effluents and potentially expose firefighters is extensive and vary with the type of fire [6]. Of primary concern, and therefore extensively assessed in firefighters’ occupational exposures, are polycyclic aromatic hydrocarbons (PAHs) due to their carcinogenic, mutagenic, and immunotoxic properties [7]. These compounds are ubiquitous byproducts of incomplete combustions of organic materials and are consistently present in fire environments.

**Figure 1:**
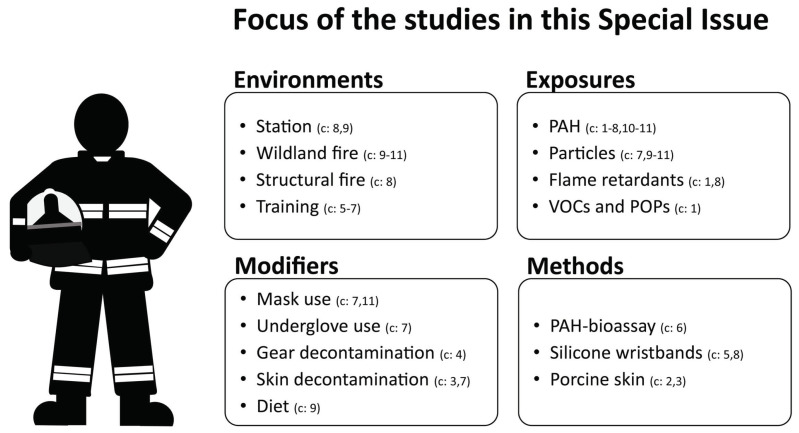
Overview of the articles published in this Special Issue. The numbers in brackets represent the list of contributions (c) in this special issue that focus on the respective areas. Bethsaida Cardona et al. (c1), Chandler Probert et al. (c2 & c3), Md Hossain et al. (c4), Paro Sen et al. (c5), Johanna Grünfeld et al. (c6), Pauline Zangl et al. (c7), William Papas et al. (c8), Filipa Esteves et al. (c9), Joana Teixeira et al., (c10) and Tanis Zanudayski et al. (c11).

This Special Issue compiles 11 articles encompassing a diverse range of research on firefighters’ exposures and health risks: one review paper, three laboratory experiments, three studies on firefighting training, and four studies focusing on real-life occupational settings, including structural, wildfire, and prescribed fire contexts. While most studies in this Special Issue focus on PAHs, several also address additional exposure concerns, including particulate matter, metal(loid)s, flame retardants, persistent organic pollutants (POPs), and volatile organic compounds (VOCs) (Figure 1).

## 2. Overview of the Published Articles

Cardona et al. (contribution 1) reviewed chemical exposures among firefighters in relation to the strength of evidence linking these substances to breast cancer or mammary tumors, identifying twelve chemicals or chemical groups of concern. These exposures come from sources such as combustion byproducts from live fires, firefighting foams, contaminated personal protective equipment (PPE), fire station environments, and diesel exhaust. The study raises a concern regarding a potential link between firefighting and breast cancer, highlighting the fact that female firefighters have historically been overlooked in firefighter occupational health research.

Three laboratory studies focused on PAH exposures. Two of these, conducted by the same research group, used a porcine skin model to study the percutaneous absorption of PAHs. In the first study, Probert et al. (contribution 2) tested the absorption under simulated sweaty firefighting conditions, demonstrating that molecular weight and solubility influenced dermal uptake. A lag time of approximately 60 min was observed, supporting the importance of skin cleansing practices to reduce PAH exposures. In the second study (contribution 3), Probert et al. assessed if ingredients from four different skin decontamination wipe solutions affected phenanthrene absorption, concluding that the wipe solutions did not enhance skin penetration, supporting its use in decontamination practices.

Hossain et al. (contribution 4) investigated key factors influencing PAH removal during the laundering of firefighter PPE. While longer presoaking durations enhanced PAH removal—especially for high-molecular-weight PAHs—detergent concentration emerged as the most critical factor in decontamination efficiency.

Sen et al. (contribution 5) used a firefighting training context to test the use of passive silicone bands for measuring PAHs, pairing them with conventional active air sampling methods. The authors demonstrated an overall good correlation between the two approaches while also discussing certain limitations.

Grünfeld et al. (contribution 6) investigated the application of a bioassay to assess PAH exposure across different firefighting training scenarios involving wood fire, gas fire, or without fire, focusing on skin deposition and urinary excretion. Both the biological method and the chemical traditional analysis showed increased skin PAHs after smoke-diving exercises with wood fire smoke, while urinary results were less consistent.

Zangl et al. (contribution 7), also focusing on firefighting training, investigated instructors’ exposure by monitoring airborne particles at stationary positions, as well as PAH levels in skin wipes and urine samples. Using video recordings, this study also assessed instructor behaviors such as wearing undergloves, using respiratory protection, and handwashing. Based on their findings, the authors propose recommendations to reduce firefighter exposure to PAHs.

Papas et al. (contribution 8), using silicone wristbands, investigated exposure to PAH and flame retardants in different locations at suburban and urban Canadian firefighter stations, as stationary measurements, and worn by firefighters deployed to structural fire events. The results indicated that PAH exposure is primarily associated with fire suppression and contamination from gear and trucks, whereas organophosphate flame-retardants are more likely linked to their use in truck interiors and electronic equipment.

Three studies investigated wildland firefighter’s exposures. Esteves et al. (contribution 9) investigated particle exposure at fire stations and biomarkers of effect in Portuguese wildland firefighters before the fire season started. The endpoints analyzed were on buccal cell samples. The particulate exposures were not associated with the biological endpoints, but full-time firefighters exhibited elevated levels of cell death endpoints in relation to other firefighters in this cross-sectional evaluation.

In another Portuguese study, Teixeira et al. (contribution 10) characterized wildland firefighters’ exposure to particulate matter during prescribed burns. Particles were collected within the firefighters’ working area and within 3 m of the fire line using a cascade impactor, and each fraction was analyzed for PAH and metal(loid) content. Fine and ultrafine particles made up the majority of the collected mass and contained the highest concentrations of PAHs and metal(loids).

Zadunayski et al. (contribution 11) explored wildland firefighters’ perception of smoke exposure and mask use in Canadian fire crews in repeated deployments. The study combined particle measurements, urinary PAH excretion, and self-reported data on smoke exposure and mask usage. While perceived smoke exposure correlated with objective measurements, it did not align with actual adherence to respiratory protection.

## 3. Conclusions

This summary of 11 recent studies highlights critical firefighter exposures, with a particular focus on PAHs and particulate matter, while also addressing exposures to flame retardants, POPs, metals and VOCs. Mitigation strategies, such as the use of skin wipes, undergloves, proper respiratory protection, and effective decontamination procedures, have demonstrated efficacy and are emphasized. Monitoring techniques, including passive silicone samplers, offer promising alternatives to traditional air sampling in firefighting exposure assessment. Improved decontamination practices, science-based guidance and targeted PPE enhancements are needed to better protect firefighter health across all operational contexts: during training, structural and wildland fire scenarios, and station environments.

## Abbreviations

The following abbreviations are used in this Editorial:

PAH	Polycyclic aromatic hydrocarbon
POP	Persistent organic pollutant
VOC	Volatile organic compound
PPE	Personal protective equipment
